# Single-cell elderly blood–CSF atlas implicates peripherally influenced immune dysregulation in normal pressure hydrocephalus

**DOI:** 10.1073/pnas.2412159122

**Published:** 2025-05-05

**Authors:** Phan Q. Duy, Emre Kiziltug, Ana B.W. Greenberg, Neel H. Mehta, Le T. Hao, Carla Fortes, Susan Mullany, Baojian Fan, Ani Manichaikul, Andrew F. Teich, Diane Chan, Seth L. Alper, Bradley T. Hyman, Steven E. Arnold, Guy M. McKhann, Matthew P. Frosch, Kristopher T. Kahle

**Affiliations:** ^a^Department of Neurosurgery, Massachusetts General Hospital, Boston, MA 02114; ^b^Department of Genome Sciences, Center for Public Health Genomics, University of Virginia, Charlottesville, VA 22903; ^c^Department of Pathology and Cell Biology, Columbia University, New York, NY 10032; ^d^Department of Neurology, Taub Institute for Research on Alzheimer’s Disease and the Aging Brain, Columbia University, New York, NY 10032; ^e^Department of Neurology, Columbia University, New York, NY 10032; ^f^Department of Neurology, Massachusetts General Hospital, Boston, MA 02114; ^g^Division of Nephrology and Department of Medicine, Beth Israel Deaconess Medical Center, Boston, MA 02115; ^h^Department of Medicine, Harvard Medical School, Boston, MA 02115; ^i^Broad Institute of Massachusetts Institute of Technology (MIT) and Harvard, Cambridge, MA 02142; ^j^Department of Neurosurgery, Columbia University, New York, NY 10032; ^k^Department of Pathology, Massachusetts General Hospital, Boston, MA 02114

## Abstract

We have generated a single-cell RNA sequencing atlas of peripheral blood and ventricular CSF in idiopathic normal pressure hydrocephalus (iNPH) patients totaling 140,207 single-cell transcriptomes. We found proinflammatory alterations in peripheral blood and CSF monocytes in iNPH patients with lower baseline cognitive function. We also identified CSF cell populations likely representing periventricular sloughing of degenerating neuroglial cells. Our findings suggest possible immune dysregulation in the blood and CSF of iNPH patients.

Idiopathic normal pressure hydrocephalus (iNPH) is an enigmatic form of hydrocephalus affecting >6% of individuals over 80 y of age (1). The pathogenesis of iNPH remains poorly understood. Here, we performed single-cell RNA sequencing (scRNAseq) of peripheral blood mononuclear cells (PBMCs) and ventricular CSF cells collected from iNPH patients.

## Methods

**Fig. 1.**
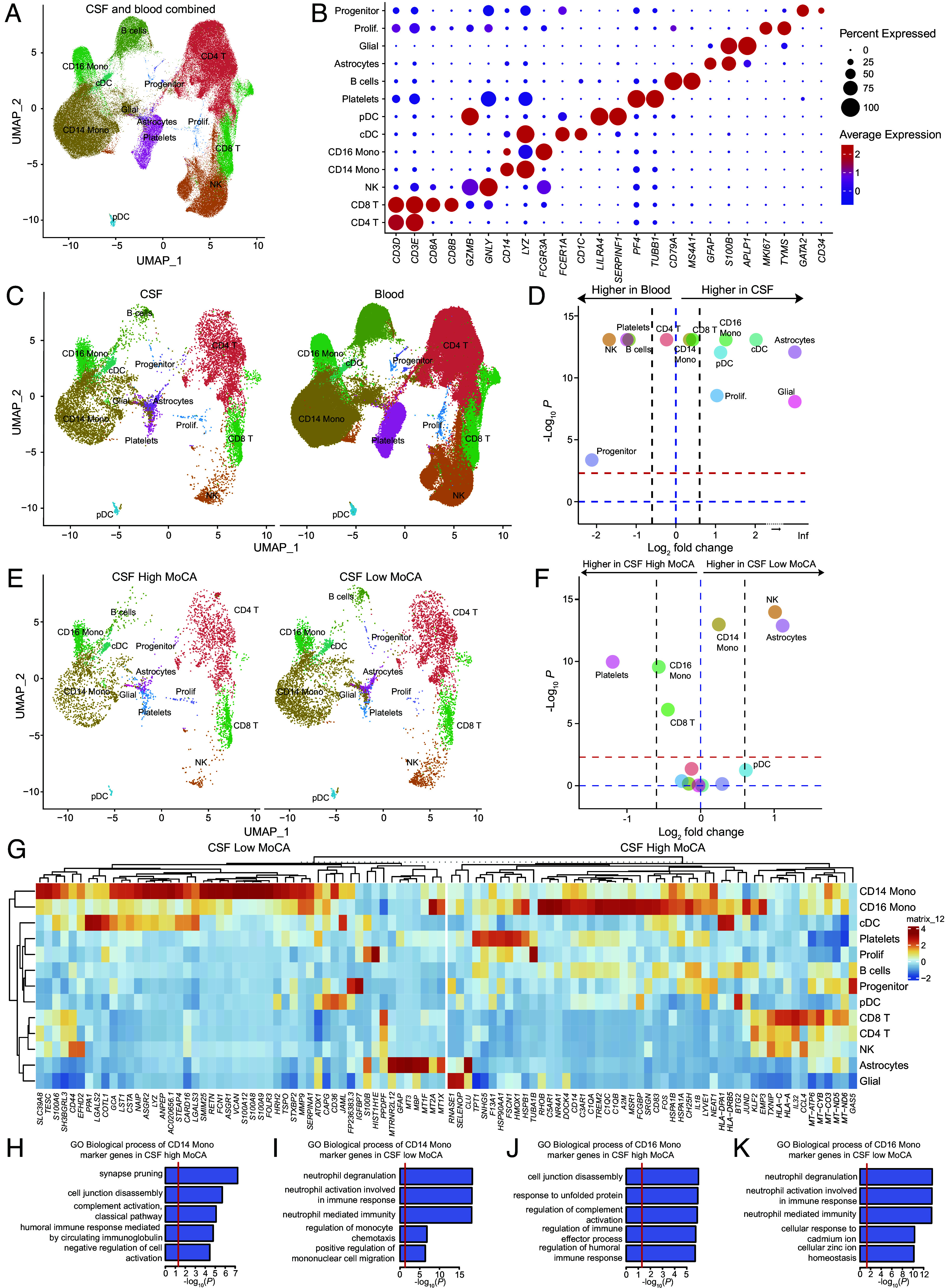
A single-cell multitissue atlas of human iNPH. (*A*) Uniform Manifold Approximation and Projection (UMAP) plot representing 13 color-coded cell clusters identified in merged single-cell transcriptomes of blood (128,027) and CSF (12,180) cells from 10 iNPH patients. Cluster names were manually assigned. (*B*) Dotpot depicting selected marker genes in blood and CSF cell clusters. Dot size encodes percentage of cells expressing the gene, color encodes the average per cell gene expression level. (*C*) UMAP plots comparing blood (*Left*) and CSF (*Right*) cell clustering. (*D*) Volcano plot depicting differences in cluster abundance in CSF compared to blood plotting fold change (log_2_) against *P* value (−log_10_) based on beta-binomial regression. Horizontal line indicates significance threshold. (*E*) UMAP plots comparing CSF cell clustering in iNPH patients with high MoCA scores (*Left*) and low MoCA scores (*Right*). (*F*) Volcano plot depicting differences in cluster abundance in the CSF of iNPH patients with high MoCA scores compared to those with low MoCA scores plotting fold change (log_2_) against *P* value (−log_10_) based on beta-binomial regression. Horizontal line indicates significance threshold. (*G*) Heatmap showing cell type–specific gene expression changes in the CSF of iNPH patients with low MoCA scores compared to those with high MoCA scores. (*H*–*K*) GO analysis (biological processes) of top marker genes for each cell cluster.

We collected ventricular CSF and blood from ten iNPH patients and processed samples to isolate CSF cells and PBMCs for scRNAseq (Fig. 1*A*; see SI Appendix, Extended Methods).

## Results

Unbiased clustering of CSF cells and PBMCs identified 13 cell types: progenitor cells (progenitor; 280 cells), proliferating cells (prolif.; 478 cells), glial cells (73 cells), astrocytes (505 cells), B cells (9,691 cells), platelets (6,639 cells), plasmacytoid dendritic cells (pDC; 581 cells), classical dendritic cells (cDC; 1640 cells), CD16^+^ monocytes (6,842 cells), CD14^+^ monocytes (35,977 cells), natural killer cells (NK; 23,337 cells), CD8^+^ T cells (11320 cells), and CD4^+^ T cells (42,844 cells) (Fig. 1 *A* and *B*). As previously observed by scRNAseq of blood and lumbar CSF (2), we identified a compartment-specific composition of immune cells in the CSF, including the enrichment of pDCs and cDCs in CSF compared to blood (Fig. 1 *C* and *D*). Unexpectedly, we also detected cells expressing markers of astrocytes and glial cells in our ventricular CSF samples that were previously unreported by scRNAseq of lumbar CSF (2, 3) (Fig. 1*C*).

We next correlated scRNAseq data with degree of neurocognitive impairment as measured by the Montreal Cognitive Assessment (MoCA). We partitioned iNPH patients into two groups: those with MoCA scores lower than 21 (low MoCA, N = 4 patients) and those with MoCA scores greater than 21 (high MoCA, N = 5 patients). The MoCA scores in the “low MoCA” group are: 19, 20, 20, 20. The MoCA scores in the “high MoCA” group are: 23, 23, 25, 27, 29. We then assessed the blood and CSF single-cell transcriptomes of these two groups to test possible correlations between molecular changes and baseline cognitive impairment. Binomial regression modelling revealed expansion of astrocytes, natural killer cells, and CD14^+^ monocytes in CSF of low MoCA patients, whereas proportions of CD8^+^ T cells and CD16^+^ monocytes were reduced (Fig. 1 *E* and *F*).

Heatmap analysis of cell type-specific gene expression showed differences in CSF cells between iNPH patients with high MoCA scores and those with low scores (Fig. 1*G*). Among CD14+ monocytes in the CSF of low MoCA iNPH patients, we found significantly increased expression of *S100A12, S100A8*, and *S100A9*, encoding pro-inflammatory calcium-binding proteins (4, 5). Gene ontology (GO) analysis of the top marker genes in each cluster revealed that CSF monocytes (CD14^+^ and CD16^+^) of low-MoCA patients expressed genes enriched in pathways related to neutrophil degranulation and neutrophil activation, whereas monocytes in the CSF of high-MoCA patients expressed genes related to synaptic pruning, cell junction disassembly, and regulation of complement activation (Fig. 1 *H*–*K*). Thus, lower baseline cognitive performance is associated with a pro-inflammatory phenotype in monocytes in iNPH.

We next compared blood scRNAseq data between high-MoCA and low-MoCA iNPH patients. Low-MoCA blood exhibited increases in the proportions of plasmacytoid dendritic cells, CD14^+^ monocytes, and CD16+ monocytes, in contrast to a decreased proportion of B cells (SI Appendix, Fig. S1 A and *B*). Blood CD14^+^ and CD16^+^ monocytes exhibited the largest differential changes in gene expression between low-MoCA and high-MoCA patients (SI Appendix, Fig. S1C). As with their matched CSF profiles, CD14^+^ peripheral monocytes of low-MoCA patients expressed pro-inflammatory signals *S100A12, S100A8*, and *S100A9* at higher levels than in high-MoCA patients (SI Appendix, Fig. S1C). CD16^+^ monocytes in low-MoCA blood exhibited increased expression of *LST1*, which encodes an adaptor protein regulating leukocyte abundance (6) (SI Appendix, Fig. S1C). Top marker genes for CD14^+^ monocytes in low-MoCA blood were enriched for terms related to lipoprotein processing, whereas CD14^+^ monocytes in high-MoCA blood were enriched for pathways related to integrin-mediated cell-cell adhesion (SI Appendix, Fig. S1 D and *E*). Marker genes for CD16^+^ monocytes in high-MoCA blood were also enriched for processes related to cell-cell adhesion, whereas top marker genes for CD16^+^ monocytes in low MoCA blood were enriched for terms related to neutrophil activation, suggesting conversion to a pro-inflammatory phenotype (SI Appendix, Fig. S1 F and *G*).

## Discussion

We have generated a single-cell transcriptomic atlas of blood and ventricular CSF from iNPH patients that revealed potential evidence of immune dysregulation in patients with lower baseline cognitive function. These data corroborate previous profiling studies of iNPH brain tissue showing microglial immune responses in association with Aβ pathology (7, 8) while also highlighting the utility of CSF and blood molecular profiling for furthering the study of iNPH pathology.

Our study provides a single-cell transcriptomic analysis of ventricular CSF, as opposed to previous studies that examined CSF obtained from the lumbar cistern (3). The lumbar cistern has long been considered the gold standard source of CSF for diagnostic workup based on the assumption that CSF has a uniform composition. Our identification of glial cells in ventricular CSF previously undescribed in spinal tap-derived CSF revealed unexpected anatomic compartmental specificity of CSF cell composition, suggesting the need for further studies of brain-derived CSF in the context of human neurological disorders.

Limitations of this study include our relatively small iNPH cohort of ten patients, which nonetheless allowed the detection of potential immune transcriptomic changes in blood and CSF. The findings motivate future investigations in expanded patient cohorts using similar unbiased molecular profiling approaches. The resulting data provide proof-of-principle for the utility of collecting blood and ventricular CSF during neurosurgical shunting, a common procedure currently indicated for a wide variety of nervous system pathologies. A similar collection strategy could be informative for cellular and molecular characterization of diseased tissues in other neurological conditions beyond iNPH for which neurosurgical shunting is also indicated.

## Supplementary Material

Appendix 01 (PDF)

## Data Availability

scRNAseq data have been deposited in GEO (GSE292141) (9).
