# Supplementary material for: Single-cell elderly blood–CSF atlas implicates peripherally influenced immune dysregulation in normal pressure hydrocephalus
Source: Proc Natl Acad Sci U S A. 2025 May 5;122(19):e2412159122. doi: 10.1073/pnas.2412159122 (PMC12087963; doi:10.1073/pnas.2412159122)
Supplement: Supplementary file 1 — Appendix 01 (PDF) [file pnas.2412159122.sapp.pdf]

## EXTENDED METHODS

Ethics statement

This study, including acquisition, and use of human samples was approved by the Institutional Review Board at Massachusetts General Hospital (MA, USA). All human tissue samples were obtained with written informed consent prior to tissue collection from participants.

Patient recruiting and tissue collection

We recruited 10 iNPH patients who underwent neurosurgical shunting for CSF diversion at the MGH neurosurgery service. These patients presented with suspected iNPH based on the presence of at least one of the classic “triad” symptoms (gait impairment, cognitive decline, and urinary incontinence) with neuroimaging demonstrating ventricular dilation. All patients underwent a CSF diversion trial in which CSF is drained by placement of a temporary lumbar drain or by a large-volume lumbar puncture tap. For patients whose temporary CSF diversion trial resulted in improvements of clinical symptoms, a ventriculoperitoneal shunt was placed for permanent CSF diversion. During the neurosurgical operation to place a shunt, we collected CSF from the ventricular compartment and peripheral blood.

Blood and CSF sample processing

PBMCs were isolated from fresh whole blood using Ficoll-Paque gradient according to manufacturer’s protocol. Cells were washed twice in 1× Dulbecco’s PBS and resuspended in 1× Dulbecco’s PBS with 0.04% bovine serum albumin ranging from 800-1200 cells/µl. Fresh CSF was kept on ice until processing. CSF was centrifuged at 1500 rpm for 10 minutes, and supernatant was aspirated to leave 50 µl for cell counting and loading. PBMC and CSF samples were counted using 4% trypan blue and a hemocytometer. Single-cell suspensions were loaded onto the Chromium Single Cell Controller using 10x Genomics Single Cell 5’ v2 kit and protocol. A target cell number of 10000 was used for PBMCs. All CSF cells were loaded. Samples were sequenced on NovaSeq S4.

Single-cell RNA sequencing and pre-processing

scRNAseq data from PBMC and CSF samples were processed according to the following steps. Sample demultiplexing and read alignment to the NCBI reference GRCh38-2020-A was completed to map reads to both unspliced pre-mRNA and mature mRNA transcripts using CellRanger version 7.0.1. The preprocessing and clustering analysis for sc/snRNA of human CSF, PBMCs, and brain was completed using Seurat (Hao et al. 2021). Cells with <200 genes, <500 unique molecular identifiers (UMIs) or >10% of mitochondrial gene origin were removed. The normalization and initial feature selection for each of the sample datasets were completed individually. The filtered matrices were normalized using SCTransform in Seurat. The experimental conditions were integrated using Seurat’s integration pipeline for PBMC and CSF datasets. For integration, 3,000 shared highly variable genes were identified using Seurat’s ‘SelectIntegrationFeatures()’ function. Integration anchors identified based on these genes were identified by canonical correlation analysis using the ‘FindIntegrationAnchors()’ function. The data were then integrated using ‘IntegrateData()’, yielding an integrated scRNAseq dataset of 128,027 PBMC and 12,180 CSF cells.

Principal component analysis (PCA) and uniform manifold approximation and projection (UMAP) dimension reduction with 40 principal components were performed. A KNN graph was constructed based on the euclidean distance in PCA space to embed cells in a graph structure. The edge weights were refined between any two cells based on the shared overlap in their local neighborhoods (Jaccard similarity) using the FindNeighbors (reduction=’pca’, dims=1:40) command with the first 40 principal components as input. For unsupervised cell clustering by the FindClusters function, the Louvain algorithm (default) was applied to iteratively group cells with the goal of optimizing the standard modularity function. For visualization purposes, the dimensionality of the datasets was further reduced to 2D embeddings using UMAP on the significant PCs via RunUMAP() functions of the Seurat package in R. Non-parameteric Wilcoxon rank sum tests were used to identify differentially expressed markers across all clusters by running FindAllMarkers (dataset, only.pos = TRUE, min.pct = 0.25, logfc.threshold = 0.25). Through Seurat, Wilcoxon rank-sum test was used with a total of 36,601 genes tested for multiple comparisons. Bonferroni Correction was used to calculated adjusted p values. Individual celltype clusters were annotated based on canonical markers from the literature (Schafflick et al. 2020). Further unsupervised sub-clustering of certain cell populations including myeloid cells, T-cells and neurons were conducted using the Louvain algorithm. The R ComplexHeatmap package was used to visualize the heatmap expression of highly variable genes. VlnPlot, FeaturePlot and DotPlot functions were used to visualize the gene expression profiles across clusters and conditions.

Cluster abundance analysis

In order to test the difference in cell counts between conditions, we used beta-binomial generalized linear model in package aod::betabin (Schafflick et al. 2020). In our generalized linear model, we set the count of the cell type of interest and the total count of cells of each experimental condition to be the response variable and the experimental condition of the cells to be the independent variable. We tested for Pearson's correlation between the frequency of each cell cluster. We adjusted the p value threshold using Bonferroni correction.

Gene Ontology enrichment analysis

The gene lists for differentially expressed genes were further studied for gene ontology, pathway and upstream transcription factor enrichment using the Enrichr R package (Kuleshov et al. 2016). The Enrichr contains a diverse and up-to-date collection of over 100 gene set libraries available for analysis and download. The databases for these analyses include gene ontologies (biological processes, cellular components, and molecular functions) and biological pathways (Wiki pathways Human and Mouse). Fisher's exact test was used with a total of 27,186 pathways from Gene Ontology Biological Processes Database (https://pubmed.ncbi.nlm.nih.gov/36866529/) were tested for comparisons. FDR Correction was used to calculate adjusted P-values. Adjusted p-value of less than 0.05 was considered significant.

References

Hao, Yuhan, Stephanie Hao, Erica Andersen-Nissen, William M. Mauck 3rd, Shiwei Zheng, Andrew Butler, Maddie J. Lee, et al. 2021. “Integrated Analysis of Multimodal Single-Cell Data.” *Cell* 184 (13): 3573-3587.e29.

Kuleshov, Maxim V., Matthew R. Jones, Andrew D. Rouillard, Nicolas F. Fernandez, Qiaonan Duan, Zichen Wang, Simon Koplev, et al. 2016. “Enrichr: A Comprehensive Gene Set Enrichment Analysis Web Server 2016 Update.” *Nucleic Acids Research* 44 (W1): W90-7.

Schafflick, David, Chenling A. Xu, Maike Hartlehnert, Michael Cole, Andreas Schulte-Mecklenbeck, Tobias Lautwein, Jolien Wolbert, et al. 2020. “Integrated Single Cell Analysis of Blood and Cerebrospinal Fluid Leukocytes in Multiple Sclerosis.” *Nature Communications* 11 (1): 247.
